# Association between fixation type and revision risk in total knee arthroplasty patients aged 65 years and older: a cohort study of 265,877 patients from the Nordic Arthroplasty Register Association 2000–2016

**DOI:** 10.1080/17453674.2020.1837422

**Published:** 2020-11-04

**Authors:** Tero Irmola, Ville Ponkilainen, Keijo T Mäkelä, Otto Robertsson, Annette W-Dahl, Ove Furnes, Anne M Fenstad, Alma B Pedersen, Henrik M Schrøder, Antti Eskelinen, Mika J Niemeläinen

**Affiliations:** a Coxa Hospital for Joint Replacement, and Faculty of Medicine and Health Technologies, University of Tampere, Tampere, Finland;; b Finnish Arthroplasty Register, National Institute for Health and Welfare, Helsinki, Finland;; c Department of Orthopaedics and Traumatology, Turku University Hospital, Turku, and University of Turku, Turku, Finland;; d The Swedish Knee Arthroplasty Register, Department of Orthopedics, Skane University Hospital, Lund, Sweden. Department of Clinical Sciences, Orthopedics, Lund University, Sweden;; e The Norwegian Arthroplasty Register, Department of Orthopaedic Surgery, Haukeland University Hospital, Bergen, Norway;; f Department of Clinical Medicine, University of Bergen, Bergen, Norway;; g Department of Clinical Epidemiology, Aarhus University Hospital. Denmark and Danish Knee Arthroplasty Registry;; h Department of Orthopaedic Surgery, Naestved Hospital, Denmark

## Abstract

Background and purpose — The population of the Nordic countries is aging and the number of elderly patients undergoing total knee arthroplasty (TKA) is also expected to increase. Reliable fixation methods are essential to avoid revisions. We compared the survival of different TKA fixation concepts with cemented fixation as the gold standard.

Patients and methods — We used data from the Nordic Arthroplasty Register Association (NARA) database of 265,877 unconstrained TKAs performed for patients aged ≥ 65 years with primary knee osteoarthritis between 2000 and 2016. Kaplan–Meier (KM) survival analysis with 95% confidence intervals (CI) and the Cox multiple-regression model were used to compare the revision risk of the fixation methods.

Results — Cemented fixation was used in 243,166 cases, uncemented in 8,000, hybrid (uncemented femur with cemented tibia) in 14,248, and inverse hybrid (cemented femur with uncemented tibia) fixation in 463 cases. The 10-year KM survivorship (95% CI) of cemented TKAs was 96% (96 − 97), uncemented 94% (94 − 95), hybrid 96% (96 − 96), and inverse hybrid 96% (94 − 99), respectively. Uncemented TKA was associated with increased risk of revision compared with the cemented TKA; the adjusted hazard ratio was 1.3 (95% CI 1.1 − 1.4).

Interpretation — Cemented, hybrid, and inverse hybrid TKAs showed 10-year survival rates exceeding 95%. Uncemented fixation was associated with an increased risk of revision in comparison with cemented fixation. As both hybrid and inverse hybrid fixation were used in only a limited number of TKAs, indicating possibility of selection bias in their favor, cemented TKA still remains the gold standard, as it works reliably in the hands of many.

Cemented fixation of total knee arthroplasty (TKA) has been regarded as the gold standard. Uncemented fixation potentially offers some advantages, such as shorter operative times and elimination of possible complications of using bone cement (Yayac et al. 2020), but also some obvious disadvantages such as higher implant costs and increased risk of revision as shown in register-based studies (Nugent et al. 2019, NJR 2019). In a study from the New Zealand Joint Registry (NZJR) uncemented TKAs had similar patient-reported outcomes but higher revision rates when compared with hybrid and cemented TKAs (Nugent et al. 2019). Survivorship between unconstrained cemented and hybrid TKAs did not differ either in the National Joint Registry for England, Wales and North Ireland (NJR) or in the Australian Orthopaedic Association National Joint Replacement Registry (AOANJRR), while uncemented TKAs showed a slightly higher risk of revision (AOANJRR 2019, NJR 2019). Conversely, a Norwegian register-based study reported better survivorship of hybrid than cemented TKAs at 11 years (Petursson et al. 2015). Further, a recent meta-analysis found no differences in either implant survivorship or clinical outcomes between uncemented and cemented fixation (Zhou et al. 2018).

We demonstrated recently that cemented TKA should be considered the gold standard in patients younger than 65 years of age even if promising survivals were detected in hybrid TKAs (Niemeläinen et al. 2020). In the current study, we assessed whether the traditional assumption of cemented TKA as gold standard still holds true also in elderly patients. We analyzed survivorships of different fixation methods in unconstrained TKA in patients aged 65 years and older based on the Nordic Arthroplasty Register Association (NARA) database.

## Patients and methods

### Study design and setting

We conducted this population-based cohort study using prospectively collected data available from the NARA knee dataset, which contains data from 4 Nordic countries (the Swedish Knee Arthroplasty Register [SKAR], the Danish Knee Arthroplasty Register [DKR], the Norwegian Arthroplasty Register [NAR], and the Finnish Arthroplasty Register [FAR]). The dataset includes only variables that all countries can deliver, currently 20 variables for knee arthroplasty (Mäkelä et al. 2019). All Swedish, Norwegian, Danish, and Finnish citizens are assigned a unique civil registration number, permitting unambiguous linkage between knee registries and other medical databases in each country. All registers have used individual-based registration of operations. Selection and transformation of the particular datasets and de-identification of the operations were performed within each national register. The anonymous data was then merged into a common dataset. Data were treated with full confidentiality, according to the rules of the respective countries.

The quality of data in the Nordic registers is high, including both 100% coverage and following completeness for primary TKA: SKAR 97%, DKR 97%, NAR 97%, FAR 96% and for revision TKA: SKAR > 95% (estimate, OR, personal communication), DKR 94%, NAR 91%, FAR 80% (NAR 2018, DKR 2019, FAR 2019, SKAR 2019). The study follows the RECORD and STROBE guidelines.

### Study population

**Figure 1.**
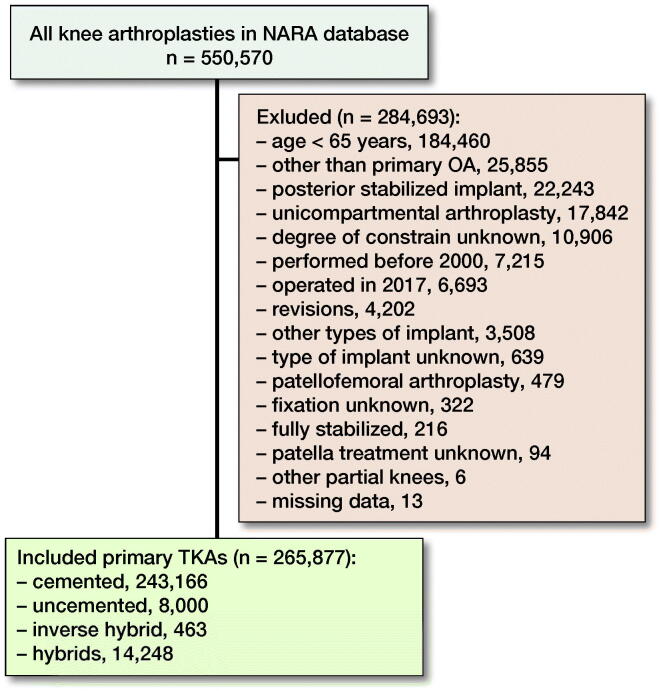
Flow chart of the study.

We included all uni- or bilateral unconstrained primary TKAs that had been implanted in patients aged 65 years or older for primary OA 2000–2016 (Figure 1). Bilaterals were both same-day bilateral and staged bilateral. Previous reports have shown that the effect of including bilateral cases in studies of hip and knee prosthesis survival is negligible (Robertsson and Ranstam 2003, Lie et al. 2004). The fixation of TKAs was divided into 4 groups: (1) cemented, (2) uncemented, (3) hybrid (uncemented femur with cemented tibia), and (4) inverse hybrid (cemented femur with uncemented tibia). The numbers of included implants and reasons for exclusions are shown in a flow chart (Figure 1).

### Outcome

The primary outcome measure was time to 1st revision, defined as removal, addition, or exchange of at least 1 of the components for any reason. Thus, the 1st revision of the index knee was the endpoint.

### Statistics

Descriptive statistics were presented as numbers (%), as mean (SD), or as median with interquartile range (IQR) based on the distribution. Kaplan–Meier (KM) survival analysis was used to assess implant survival probability with 95% confidence intervals (CI) of the TKA fixation. Groups with less than 40 knees at risk are not presented in the tables.

We used multivariable Cox proportional hazard regression analysis to compare the survival between different fixation types adjusting for confounding variables (hazard ratios). Fixation type was used as the primary dependent variable and all analyses were adjusted for potential confounders such as sex, country, patellar resurfacing, and age. Age was included in the model as continuous variable whereas the others were categorical. Sensitivity analyses were performed for subgroups based on age (65 − 75 years of age and older than 75), sex, and NexGen TKA model. Because of the obvious risk for case-mix bias, an additional sensitivity analysis was conducted for patients operated on with NexGen TKAs. We examined violations of proportional hazard (PH) assumptions by evaluating the correlation of scaled Schoenfeld residuals with time. In addition, the correlation of scaled Schoenfeld residuals and log–log survival curves were inspected visually to evaluate the PH assumptions. Violations of PH assumptions were handled by constructing a time-stratified model (Zhang et al. 2018). Thus, the time-stratification was conducted for a unique set of variables based on the Schoenfeld residuals and log–log survival curves of the particular model. Correlations of Schoenfeld residuals with time were repeatedly evaluated to ensure that the non-proportionality was fixed. Statistical analyses were performed using R 3.6.2 (R Foundation for Statistical Computing, Vienna, Austria), with packages survival, survminer, and tidyverse.

### Ethics, funding, and potential conflicts of interest

Formal approval for the study was granted by the ethical approval process of each national register. Permission numbers from each country are: the Danish Data protection agency (1-16-02-54-17), Denmark, the National Institute of Health and Welfare (Dnro THL/1743/.5.05.00/2014), Finland, the Norwegian Data Inspectorate (ref 24.1.2017: 16/01622-3/CDG), Norway, and the Ethics Board of Lund University (LU20-02), Sweden. This work was supported by the competitive research funds of Pirkanmaa Hospital District, Tampere, Finland, representing governmental funding. The authors have no conflicts of interests to declare.

## Results

**Figure 2.**
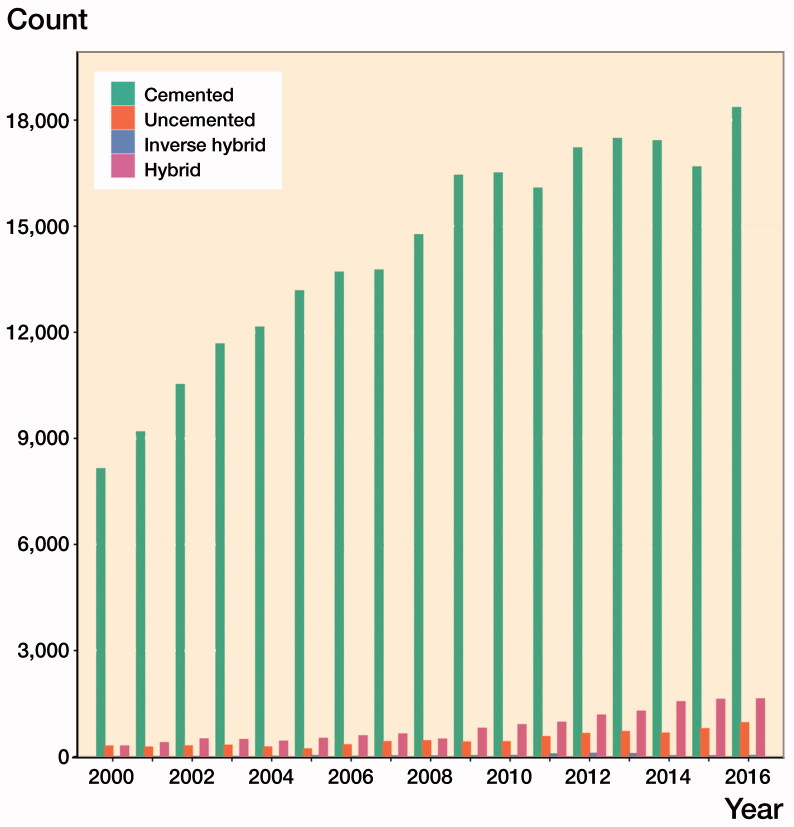
Number of TKAs with different fixation methods.

Cemented fixation was used in 92% of all TKAs, although there was some variation between the countries: Sweden (98%), Finland (98%), Norway (79%) to Denmark (73%). Hybrid fixation was used in 5% of all cases: Denmark (21%), Norway (15%), Finland (0.4%), and Sweden in 36 cases (0.0%) (Table 1). The total number of TKAs performed annually increased notably (102%) between the years 2000 (n = 8,733) and 2009 (n = 17,668) but remained relatively stable after that. The use of hybrid fixation increased by 104% between the years 2009 (n = 798) and 2016 (n = 1,631) (Figure 2). The TKA models varied between countries without a common trend and the most commonly used TKA models are shown in the Table 2 (see Supplementary data). NexGen, PFC, and Triathlon were the most commonly used models within the fixation concepts (Table 3, Supplementary data). The patella was resurfaced in 56,596 TKAs (22%) and uncemented patellar buttons were used in only 371 (0.1%) of the TKAs. There were differences between the countries when considering proportion of patellar resurfacing, from Norway (3%), Sweden (6%), and Finland (18%), to Denmark (79%) (Table 4, Supplementary data). In the subgroup of NexGen TKAs, the patella was resurfaced in 12,160 (18%) TKAs, and an uncemented patellar button was used in only 14 knees.

**Table 1.** Demographic data. Values are count (%) unless otherwise specified

Factor	Uncemented	Inverse hybrid	Hybrid	Cemented
TKAs	8,000 (3.0)	463 (0.2)	14,248 (5.4)	243,166 (92)
Mean age (SD)	73 (5.5)	73 (5.6)	74 (5.7)	74 (5.7)
Men, %	43	39	37	36
TKAs per country				
Finland	1,204 (1.5)	340 (0.4)	307 (0.4)	80,195 (98)
Norway	2,017 (6.2)	15 (<0.1)	4,793 (15)	25,900 (79)
Sweden	1,955 (1.8)	22 (<0.1)	36 (<0.1)	104,863 (98)
Denmark	2,824 (6.4)	86 (0.2)	9,112 (21)	32,208 (73)

**Table 5.** Unadjusted Kaplan–Meier (KM) 10- and 15-year survival rates with 95% confidence intervals (CI) for uncemented, inverse hybrid, hybrid, and cemented TKA

			At 10 years		At 15 years	
	No. of	No. of	No. at	KM survival	No. at	KM survival
Type of fixation	knees	revisions	risk	rate (%)(CI)	risk	rate (%)(CI)
Uncemented	8,000	321	1,271	94 (94–95)	201	93 (92–94)
Inverse Hybrid	463	11	40	96 (94–99)	–	–
Hybrid	14,248	423	2,078	96 (96–96)	254	94 (93–95)
Cemented	243,166	6,767	50,845	96 (96–97)	6,594	96 (95–96)

**Table 6.** Multivariate Cox regression with adjusted hazard ratios (aHR) and 95% confidence intervals (CI)

	Age ≥ 65 years	Age 65–75 years	Age > 75 years
Type of fixation	aHR **^a^** (CI)	aHR **^b^** (CI)	aHR **^c^** (CI)
Uncemented	1.3 (1.1–1.4)	1.1 (0.9–1.5)	1.4 (1.1–1.7)
Inverse hybrid	0.8 (0.4–1.4)	0.9 (0.4–1.8)	1.1 (0.4–2.9)
Hybrid	1.0 (0.9–1.1)	1.5 (1.1–1.7)	0.9 (0.7–1.0)
Cemented	1.0 Reference	1.0 Reference	1.0 Reference

Hazard ratios adjusted by age, sex, patellar resurfacing, and nation —age, sex, and nation as time-dependent coefficients divided into time intervals of:

**^a^** 0.1, 0.3, 0.5, 1.5, 3.5, and 6 years.

**^b^** 0.1, 0.5, 1.0, 3.5, and 6 years.

**^c^** 0.1, 1.0, 3.5, 8, and 10 years.

**Table 9.** Multivariate Cox regression of patients in NexGen subgroup with adjusted hazard ratios (aHR) and 95% confidence intervals (CI)

Type of fixation	aHR **^a^** (CI)
Uncemented	1.1 (0.8–1.5)
Inv hybrid	0.9 (0.4–1.8)
Hybrid	1.3 (1.1–1.6)
Cemented	1.0 Reference

**^a^** Adjusted by age, sex, patellar resurfacing, and nation ­—age, sex, and nation as time-dependent coefficients divided into time intervals of 0.2, 1.5, 3.5, and 6 years.

Of the 265,877 TKAs, altogether 7,522 underwent revision after median follow-up time of 5.5 years. The median follow-up time was 5.8 years for cemented, 4.7 years for uncemented, 4.7 years for inverse hybrid, and 4.2 years for hybrid TKA. Between the fixation groups, there were marginal differences in the proportion of men, ranging from 36% in the cemented to 43% in the uncemented group (Table 1).

**Figure 3.**
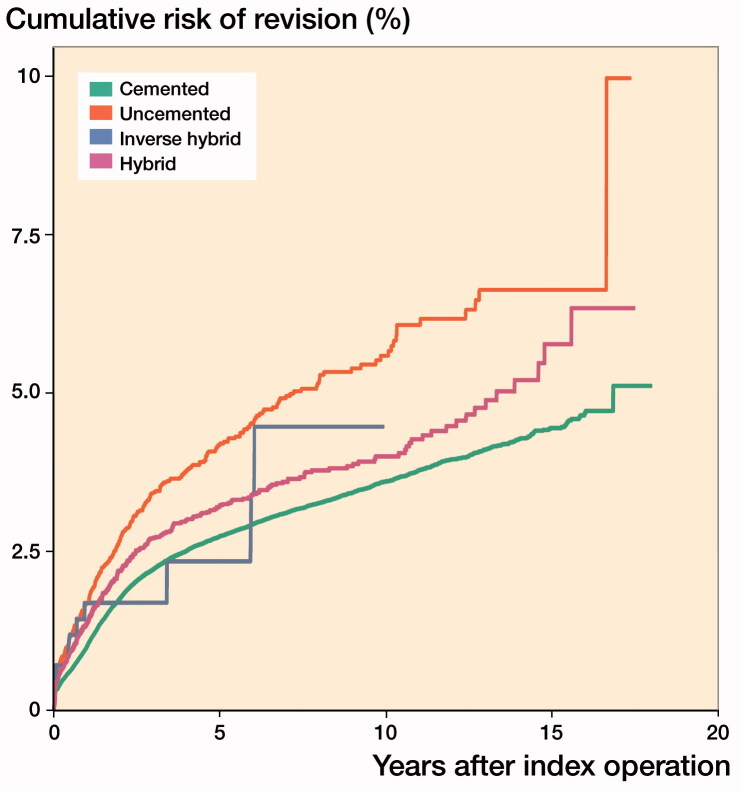
Unadjusted Kaplan–Meier cumulative risk of revision by fixation type in patients > 65 years of age (stopping rule, n = 40).

KM-based 10-year survival rates were: cemented 96%, inverse hybrid 96%, hybrid 96%, and uncemented 94%. Due to low numbers, the 15-year survival rate of inverse hybrid was not reliable, yet the 15-year survival rates for other fixation methods were: cemented 96%, hybrid 94%, uncemented 93% (Table 5, Figure 3).

Uncemented fixation evinced an increased risk of revision compared with the cemented TKA in the adjusted Cox regression analysis (HR 1.3) (Table 6). We found no differences in the risk of revision between the hybrid or inverse hybrid and the cemented TKAs. The additional Cox regression analyses were conducted for 2 different age groups: 65 − 75 years of age and older than 75 years of age (Table 6). In patients aged 65 − 75 years, the risk of revision with hybrid TKAs was increased in comparison with the cemented reference group (HR 1.5) (Table 6). In patients older than 75 years, there was an increased risk of revision with uncemented fixation (HR 1.4) (Table 6).

Most of the TKAs in the inverse hybrid group were NexGen (82%) (Table 3, Supplementary data)). Cemented fixation was used in 91% of the NexGen TKAs (Table 7, Supplementary data). Because of the obvious risk for case-mix bias, an additional sensitivity analysis was conducted for patients operated on with NexGen TKAs. In this sensitivity analysis, 7-year survival rates of different fixations were in descending order: cemented 98%, inverse hybrid 97%, uncemented 96%, and hybrid 95%. At 10 years, survival rate was available only for cemented NexGen TKAs (98%) (Table 8). An increased risk of revision was found for hybrid NexGen TKAs as compared with the cemented NexGen TKAs (HR 1.3). The risk of revision for uncemented and inverse hybrid TKAs was comparable to cemented TKAs (Table 9).  

**Table 8.** Unadjusted Kaplan–Meier 7- and 10-year survival rates with 95% confidence intervals (CI) are presented for uncemented, inverse hybrid, hybrid, and cemented TKA in the NexGen subgroup

			At 7 years		At 10 years	
	No. of	No. of	No. at	KM survival	No. at	KM survival
Type of fixation	knees	revisions	risk	rate (%)(CI)	risk	rate (%)(CI)
Uncemented	1,976	61	164	96 (94–97)	–	–
Inverse Hybrid	379	8	85	97 (94–99)	–	–
Hybrid	3,887	133	275	95 (94–96)	–	–
Cemented	61,376	1,191	16,858	98 (98–98)	6,982	97 (97–97)

## Discussion

This multinational register-based study revealed that cemented fixation was used in the vast majority of the TKAs (91%) in the Nordic countries among elderly patients. Cemented, hybrid, and inverse hybrid TKAs all evinced acceptable 10-year survival rates exceeding 95% in patients aged 65 years and older. Uncemented fixation was associated with increased risk of revision compared with cemented fixation.

The population is aging, and patients aged 65 years or more still contribute to most of the total incidence of knee arthroplasty (Niemeläinen et al. 2017), meaning that most TKAs will be performed in elderly patients. In our study the vast majority of TKAs were cemented. Cemented fixation is also the most commonly performed type of knee replacement in arthroplasty registers (AOANJRR 2019, NJR 2019, NZJR 2019). Conversely, the NZJR showed that the usage of uncemented fixation had increased during the last 3 years (NZJR 2019). In our study, the small increase in the use of hybrid fixation was mainly seen in Denmark and Norway between 2009 and 2016.

All fixation methods evinced acceptable 10-year survival rates in patients aged 65 years and older. Cemented and hybrid TKAs still showed good survivorship at 15 years. Uncemented TKAs had the lowest survivorship. These findings are in line with the majority of the previous literature. Based on the AOANJRR, the cumulative 15-year revision rate of minimally stabilized TKA was lower with cemented fixation compared with uncemented and lowest with hybrid fixation (AOANJRR 2019). The NJR (2019) and the NZJR (2019) reported the same trend among 65–74-year-old patients. Moreover, the revision rate of patients older than 75 years with cemented TKAs was lower than with uncemented and hybrid TKAs in the NJR and slightly higher with hybrid compared with cemented and uncemented TKAs in the NZJR. A higher risk of revision in cemented TKAs compared with hybrid TKAs at 11 years was noted in a Norwegian registry-based study, but after exclusion of a high-volume hospital the difference was no longer statistically significant. Of most obvious concern is that the reported result involved only 1 prosthesis brand (Petursson et al. 2015).

The risk of revision with hybrid TKAs was increased in comparison with cemented TKAs in the age group 65 − 75 years in our study. This is contrary to annual reports from the AOANJRR (2019), NJR (2019) and NZJR (2019), where hybrid TKAs are not worse than cemented. The risk of revision was increased with uncemented fixation in patients aged above 75 years. In this age group uncemented and hybrid fixation showed slightly increased risk of revision in the NJR annual report of 2019. In New Zealand there was no difference in the revision risk between cemented and uncemented fixation, but this was slightly higher with hybrid fixation (NZJR 2019).

Some studies report contradictory results on the association of fixation on TKA outcomes. In a randomized controlled trial comparing uncemented and cemented fixation in TKA (PFC), the authors reported no differences in revision rates and survival between the cemented and uncemented TKAs with mean follow-up of 9 years (Baker et al. 2007). When interpreting the results of that study, it is important to keep in mind that it was a single-surgeon series from a clinic with the same prosthesis design. Similarly, Zhou et al. (2018) found no differences between uncemented and cemented TKAs in implant survivorship and clinical outcomes in their systematic review and meta-analysis consisting of 409 uncemented and 403 cemented TKAs. There was a wide range in the average length of follow-up among the trials and population characteristics like mean age were different between the trials, which may have affected the results.

As affirmed earlier, NexGen covered the majority (82%) of the TKAs in the inverse hybrid group, and 87% of these NexGen TKAs had been used with TM tibial components, which are known to have good results (Niemeläinen et al. 2014). We tried to grasp the obvious possibility of selection bias by conducting a sensitivity analysis including only NexGen TKAs (Tables 8 and 9). In that analysis we found similar mid-term survival rates or Cox-adjusted revision risks between inverse hybrid and cemented NexGen TKAs. Further, hybrid fixation showed an increased risk for revision in this NexGen subgroup. Similar increased risk for revision with hybrid fixation was also seen in the TKAs in the age group 65–75 years. Thus, the more expensive uncemented or hybrid/inverse hybrid versions did not provide the older age group with any advantage over cemented fixation in the 10-year follow-up of NexGen TKAs.

We acknowledge certain strengths and limitations in our study. The major strength is the unique collaboration of 4 national registers in the creation of a multinational dataset comprising a high number of non-selected TKAs, reflecting the real-world outcomes of TKA. There are also some limitations in our study. First, these results concern TKA concepts, not single components and their fixation. It must be noted that there were clearly fewer patients in the alternative fixation groups as compared with the cemented reference group. This may have caused some selection bias, and in this case it might have favored concepts other than cemented fixation. Further, especially inverse hybrid fixation, but also hybrid fixation to some extent, had another advantage over cemented fixation in our study setting. In the inverse hybrid group, 82% were NexGen TKAs and more than 80% approximately of the inverse hybrid NexGen TKAs utilized TM monoblock tibial components (an estimate from national registers’ data), which are known to have good long-term results (Kim et al. 2012, Niemeläinen et al. 2014, Robertsson et al. 2020). What is more, in Finland NexGen inverse hybrid TKAs (with TM tibial component) have been performed in only 3 hospitals, one of which is a high-volume specialized center (Niemeläinen et al. 2014). In the hybrid group, 3 TKA designs with a good track record (PFC, NexGen, Profix) comprised 75% of all TKAs. Thus, the result of inverse hybrids should be interpreted with caution. Second, revision as the only outcome of interest also has several disadvantages. Revision is a surgeon and patient-dependent outcome and decision regarding which knee symptoms require revision is mostly subjective and may vary. Of course, the decision to reoperate is shared and the patient has to agree. With the exception of prosthetic joint infection and periprosthetic fracture, other indications for knee revision are less clear and less objective. Also, differences in proportions of patellar resurfacing in each country may have some role, especially on a patient’s risk of secondary patellar resurfacing. Third, completeness of revision TKA is lower in FAR than in other Nordic arthroplasty registries. In Finland, 20% of revisions are missing from the FAR database when compared with the National Patient Discharge Register. Finally, the median follow-up time of 5.5 years is rather short when evaluating the revision rate for TKA.

In conclusion, cemented TKA deserves the status of gold standard in TKA irrespective of the patients’ age. The advantages of cemented TKA are even more pronounced with increasing patient age. Even though hybrid/inverse hybrid versions of the well-performing contemporary TKA designs provided older patients with a good mid-term outcome, these results do not support systematic use of these more expensive components in TKA for older patients. Thus, for patients aged 65 years and older, cemented TKA is still the method of choice.

## Supplementary Material

Supplemental Material

## Supplementary data

Tables 2–4 and 7 are available as supplementary data in the online version of this article, http://dx.doi.org/10.1080/17453674.2020.1837422
