# Supplementary material for: Association between fixation type and revision risk in total knee arthroplasty patients aged 65 years and older: a cohort study of 265,877 patients from the Nordic Arthroplasty Register Association 2000–2016
Source: Acta Orthop. 2020 Nov 4;92(1):91–6. doi: 10.1080/17453674.2020.1837422 (PMC7919873; doi:10.1080/17453674.2020.1837422)
Supplement: Supplemental Material [file IORT_A_1837422_SM1540.pdf]

Supplementary data

Table 2. The 5 most commonly used TKA designs in each country 2000–2016

Norway		Sweden		Finland		Denmark	
LCS	10,208	NexGen	34,841	Triathlon	23,591	PFC Sigma	16,455
PROFIX	8,670	PFC Sigma	32,374	Nexgen	15,509	NexGen	10,614
NexGen	6,655	AGC	10,751	PFC Sigma	14,550	AGC	9,161
PFC Sigma	1,736	Vanguard	7,212	Duracon	11,897	Vanguard	4,681
Duracon	1,429	Triathlon TKA	7,114	Vanguard	6,109	Advance	1,589

Table 3. The 5 most commonly used TKA designs in the fixation concepts 2000–2016

Cemented		Uncemented		Hybrid		Inverse hybrid	
NexGen	61,377	NexGen	1,976	PFC Sigma	4,531	NexGen	379
PFC Sigma	32,372	Triathlon TKA	1,574	NexGen	3,887	Vanguard	34
Triathlon	24,937	PFC Sigma	1,249	PROFIX	2,266	PFC Sigma	11
AGC	19,356	Duracon	619	LCS	874	Triathlon TKA	10
Duracon	18,468	LCS	609	Advance	487	PROFIX	8

Table 4. Proportions of patellar resurfacing, no. of TKAs (%) in primary TKA in each country 2000–2016

Factor	Norway	Sweden	Finland	Denmark	Total
Resurfaced	1,002 (3.1)	6,724 (6.3)	14,552 (17.7)	34,788 (78.7)	57,066 (21.4)
No resurfacing	31,723 (96.9)	100,152 (93.7)	67,494 (82.3)	9,442 (21.3)	208,811 (78.5)

Table 7. Proportions of different fixation methods in the NexGen subgroup 2000–2016

Factor	Norway	Sweden	Finland	Denmark	Total	%
Cemented	5,908	34,796	14,511	6,161	61,376	90.8
Hybrid	501	2	8	3,376	3,887	5.7
Uncemented	244	39	655	1,038	1,976	2.9
Inverse hybrid	2	4	334	39	379	0.6
